# Association of hospice utilization and publicly reported outcomes following hospitalization for pneumonia or heart failure: a retrospective cohort study

**DOI:** 10.1186/s12913-017-2801-3

**Published:** 2018-01-09

**Authors:** Soowhan Lah, Emily L. Wilson, Sarah Beesley, Iftach Sagy, James Orme, Victor Novack, Samuel M. Brown

**Affiliations:** 1 Center for Humanizing Critical Care, Intermountain Healthcare, Murray, UT USA; 2 Pulmonary and Critical Care Medicine, Intermountain Medical Center, Murray, UT USA; 3 Pulmonary and Critical Care Medicine, University of Utah School of Medicine, Salt Lake City, UT USA; 4 Clinical Research Center, Soroka University Medical Center, Beersheba, Israel; 5 Faculty of Health Sciences, Ben-Gurion University of the Negev, Beersheba, Israel; 6 Beth Israel Deaconess Medical Center and Harvard Medical School, Boston, MA USA; 7 Shock Trauma ICU, Intermountain Medical Center, 5121 S. Cottonwood Street, Murray, UT 84107 USA

**Keywords:** Hospice, Palliative care, Outcome measures, Moral hazard, Policy analysis

## Abstract

**Background:**

The Center for Medicare and Medicaid Services (CMS) and the Hospital Quality Alliance began collecting and reporting United States hospital performance in the treatment of pneumonia and heart failure in 2008. Whether the utilization of hospice might affect CMS-reported mortality and readmission rates is not known.

**Methods:**

Hospice utilization (mean days on hospice per decedent) for 2012 from the Dartmouth Atlas (a project of the Dartmouth Institute that reports a variety of public health and policy-related statistics) was merged with hospital-level 30-day mortality and readmission rates for pneumonia and heart failure from CMS. The association between hospice use and outcomes was analyzed with multivariate quantile regression controlling for quality of care metrics, acute care bed availability, regional variability and other measures.

**Results:**

2196 hospitals reported data to both CMS and the Dartmouth Atlas in 2012. Higher rates of hospice utilization were associated with lower rates of 30-day mortality and readmission for pneumonia but not for heart failure. Higher quality of care was associated with lower rates of mortality for both pneumonia and heart failure. Greater acute care bed availability was associated with increased readmission rates for both conditions (*p* < 0.05 for all).

**Conclusions:**

Higher rates of hospice utilization were associated with lower rates of 30-day mortality and readmission for pneumonia as reported by CMS. While causality is not established, it is possible that hospice referrals might directly affect CMS outcome metrics. Further clarification of the relationship between hospice referral patterns and publicly reported CMS outcomes appears warranted.

**Electronic supplementary material:**

The online version of this article (10.1186/s12913-017-2801-3) contains supplementary material, which is available to authorized users.

## Background

In 2002 the Center for Medicare and Medicaid Services (CMS) and the Hospital Quality Alliance (HQA) began work on the evaluation and reporting of hospital performance on various process and outcome measures [1]. The resulting Hospital Compare website began reporting 10 core process measures in 2005; better performance on these process measures was correlated with a small improvement in risk-adjusted mortality [2]. Reporting of hospital-level 30-day mortality and readmission rates for acute myocardial infarction, heart failure and pneumonia soon followed.

Prior to reporting these outcomes, internal discussion at CMS addressed the matter of how to handle the question of hospice status [3]. The concern was that exclusion of all hospice patients might introduce a moral hazard, that is, an incentive to prematurely enroll high-risk patients in hospice during their hospital course in order to reduce reported mortality rates. Ultimately, the 30-day mortality rates reported beginning in 2008 excluded patients who were enrolled in hospice prior to or on the first hospital day [4]. No such adjustment was made when reporting of 30-day readmission rates began in 2010, and patients enrolled in hospice were included in these outcome measures [5].

Whether or not these policies have been successful in eliminating systemic bias related to hospice utilization is not known. However, there exist plausible mechanisms by which hospice utilization could lead to differences in outcome measures (both mortality and readmission rates). As 30-day mortality rates exclude patients previously enrolled in hospice, it is possible that hospitals with a higher proportion of patients enrolled in hospice might have lower 30-day mortality rates because of the exclusion of those hospice patients. CMS performs risk-adjustment of these 30-day mortality rates (based upon data abstracted from billing codes) [6] and it is possible that if patients enrolled in hospice have an elevated mortality risk not captured by that risk adjustment, the selective exclusion of those high-risk patients could artifactually reduce the reported 30-day mortality rates by hospitals with a high proportion of high-risk (hospice-enrolled) patients.

For hospital-level readmission rates, if patients on hospice are less likely to be readmitted, then hospitals with a larger proportion of hospice patients may demonstrate lower readmission rates. Both of these possible effects may be wholly independent of the quality of medical care provided.

Using the hospital as the unit of study, we obtained data regarding hospice utilization from the Dartmouth Atlas [7] and CMS-reported outcomes from Hospital Compare [8] in order to evaluate the question of whether the amount of hospice utilization had any association with the mortality and readmission rates reported by CMS for US hospitals. Mortality and readmission rates for pneumonia and heart failure were chosen as outcomes for their potential relevance to end-of-life decision making [9, 10]; acute myocardial infarction was not included given its lower probability of relevance to end-of-life decision making.

## Methods

### Data

The Center for Medicare and Medicaid Services (CMS) has reported United States hospital-level 30-day mortality rates for pneumonia, acute myocardial infarction and heart failure since 2008, and 30-day readmission rates for those same conditions since 2010 [1].CMS uses administrative claims data to adjust for the variation in acuity and comorbid conditions encountered by different hospitals [11], an approach that has been shown to be comparable to models built using clinical data [6]. 30-day risk-standardized mortality and readmission rates for these conditions are reported as percentages.

As hospice utilization is not reported by CMS, a separate data source was required. The Dartmouth Atlas provides hospital-level data on hospice utilization among patients enrolled in traditional fee-for-service Medicare (Parts A and B) who were hospitalized at some point during the final 2 years of their life with a diagnosis code of one of nine chronic conditions (Additional file 1: Table S1) [12]. Included in this data is the number of days spent in hospice per decedent, percentage of decedents enrolled in hospice and Medicare spending broken down by category, in addition to a variety of other healthcare utilization metrics. Patients with admissions at more than one hospital were assigned to the hospital they used most frequently [12]. The Dartmouth Atlas is a project of the Dartmouth Institute, a center for research, education and policy advocacy [13].

As our unit of measure was the hospital, we obtained data from 2012, the most recent year in which data from both the Dartmouth Atlas and CMS was available (downloaded from their respective websites) [7, 8]. Measures of hospice utilization (Dartmouth Atlas) and 30-day mortality or readmission (CMS) were merged at the hospital level.

This study analyzed publicly available data and did not involve any patient-level information, it was exempted (as per 45 CFR 46.101(b)(4)) from review by the Intermountain Healthcare Institutional Review Board.

### Definitions and other variables

Hospice utilization is reported by the Dartmouth Atlas as either percentage of decedents enrolled in hospice or the number of days spent on hospice per decedent for a given hospital. As the percentage of decedents enrolled in hospice could be identical for two hospitals with very early or very late enrollment in hospice, we chose to use days spent on hospice per decedent as it reflects both proportional and temporal hospice utilization.

Several prespecified covariates were included in the model to reduce potential sources of confounding. A composite quality measure was created from process of care measures reported by each hospital to CMS using standard methodology and expressed as a percentage (i.e., mean performance on publicly reported outcome measures, e.g. door-to-balloon time of <90 min for ST-elevation myocardial infarction, or perioperative antibiotics, a full list of which is available in Additional file 1: Table S2) [14]. Median household income of each hospital zip code as well as population density were obtained from United States (US) census data [15, 16]. Acute care beds per 1000 residents in each hospital service area was obtained from the Dartmouth Atlas [7]. Hospitals were divided into the four primary regions of the country as defined by the US Census Bureau, with the Midwestern region set as the reference region for its central location and central-leaning socioeconomic characteristics [17].

### Statistical methods

In order to evaluate the possibility of non-linear associations, we performed multivariable quantile regression [18] to describe the association between hospital-level hospice utilization and 30-day mortality and readmission rates for pneumonia and heart failure in 2012 as reported by CMS. As opposed to ordinary regression, which models the mean of an outcome, quantile regression allows modeling of various quantiles; we modeled the 25th, 50th (median) and 75th percentiles. (Further details regarding quantile regression are available in the Additional file 1).

An estimate of effect size regarding hospice utilization and the publicly reported outcome was made by taking the mean of the three regression lines and then calculating the difference in outcome rates for hospitals in the 25th percentile of hospice utilization and those in the 75th percentile of hospice utilization. For ease of interpretation, this change in publicly reported outcome rate was expressed as a function of change in relative percentile of performance.

A linear multilevel regression was also performed as a sensitivity analysis, with random intercepts grouped by region (allowing for variance in baseline hospice utilization rates by region), and all other above variables as fixed effects. Standard assumptions of linear mixed models (i.e. normal distribution, no within-group correlation in the covariance structure) were applied.

We performed all analyses in the R Statistical Package, version 3.1.3 [19].

## Results

The total number of United States hospitals with data from both CMS (30-day mortality and readmission rates, *N* = 4477) and the Dartmouth Atlas (mean days on hospice per decedent, *N* = 2503) was 2196.

Each hospital reported the mean number of days spent in hospice per decedent during the last 6 months of life. The median for reporting hospitals was 19.8 days with an interquartile range (IQR) of 15.2–24.6. The percentage of patients enrolled in hospice at time of death was also reported; the median of reporting hospitals was 52.0% (IQR 43.6–59.7). Additional data is in Additional file 1: Table S3, listed by quantiles of hospice utilization.

Median values of CMS-reported outcomes for pneumonia: 30-day mortality rates were 12.0% (IQR 10.7–13.1) and 30-day readmissions rates were 18.5% (IQR 17.4–19.8). For heart failure, 30-day mortality rates were 11.5% (IQR 10.4–12.7) and 30-day readmission rates were 24.7% (IQR 23.4–26.1).

### Pneumonia 30-day mortality rates

**Fig. 1:**
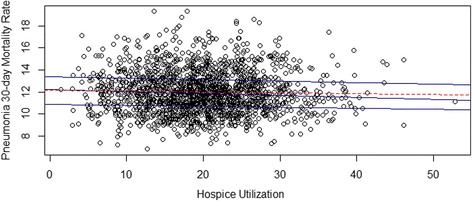
Scatterplot of 30-day pneumonia mortality rates and mean number of days in hospice per decedent; quantile regression lines are blue (25th, median and 75th percentiles), the ordinary least squares line is dotted red

Higher rates of hospice utilization were associated with decreased 30-day pneumonia mortality for hospitals in the 25th (*p* = 0.02), 50th (*p* < 0.001) and 75th percentiles (*p* = 0.04), after controlling for covariates, including quality of care scores. Hospital 30-day pneumonia mortality rates are presented as a scatterplot in Fig. 1, which illustrates the utility of quantile regression (25th, 50th and 75th percentiles) to better model non-linearities in the data.

Higher quality of care scores, higher median income and greater population density were also associated with lower rates of 30-day mortality for all 3 quantiles (*p* < 0.05), as reported in Table 1. Hospitals in the southern region of the US had higher 30-day mortality rates for pneumonia (Table 1) than those in the reference group (Midwest).

**Table 1:** Quantile Regression of Pneumonia 30-day Mortality Rates

	25th percentile	50th percentile	75th percentile
Hospice days per decedent	**−0.016**	**−0.028**	**−0.023**
Quality of care score	**−0.047**	**−0.036**	**−0.064**
Acute care hospital beds per 1000 residents	−0.0099	−0.0010	−0.045
Median income (in thousands, USD)	**−0.0087**	**−0.0010**	**−0.0012**
Population density (in thousands, per square mile)	**−0.041**	**−0.032**	**−0.033**
Northeast region**	−0.084	−0.21	−0.14
Southern region**	**0.40**	**0.28**	**0.38**
Western region**	0.15	0.11	0.29

^*^Values for which p < 0.05 are bolded

^**^Reference region: Midwest

### Pneumonia 30-day readmission rates

Higher rates of hospice utilization were consistently associated with lower 30-day pneumonia readmission rates for hospitals in the 25th (*p* = 0.02), 50th (*p* = 0.002) and 75th percentiles (*p* = 0.04), after controlling for covariates, including quality of care metrics (Table 2).

**Table 2:** Quantile Regression of Pneumonia 30-day Readmission Rates

	25th percentile	50th percentile	75th percentile
Hospice days per decedent	**−0.015**	**−0.021**	**−0.017**
Quality of care	−0.0049	**0.014**	**0.035**
Acute care hospital beds per 1000 residents	**0.25**	**0.29**	**0.50**
Median income (in thousands, USD)	**0.0073**	**0.0034**	0.0037
Population density (in thousands, per square mile)	**0.036**	**0.040**	**0.048**
Northeast region**	0.26	0.14	0.20
Southern region**	0.25	0.13	0.26
Western region**	**−0.39**	**−0.54**	**−0.42**

^*^Values for which p < 0.05 are bolded

^**^Reference region: Midwest

A greater number of acute care beds available per 1000 residents and higher population density were associated with higher readmission rates (*p* < 0.05) in all 3 quantiles. Higher quality of care scores were also associated with higher readmission rates in the 50th and 75th percentiles, while higher median income was associated with higher readmission rates in the 25th and 50th percentiles (*p* < 0.05). The western US had lower readmission rates in comparison to the reference group (Midwest).

### Heart failure 30-day mortality rates

Increased hospice utilization was associated with higher 30-day mortality in the 25th percentile (*p* = 0.01), but not in the 50th and 75th percentiles (Additional file 1: Table S4). Higher quality of care scores were associated with decreased 30-day mortality rates for heart failure in all 3 quantiles (*p* < 0.05). Higher population density was associated with lower 30-day mortality rates in all 3 quantiles (*p* < 0.05) and higher median income was associated with lower 30-day mortality rates in 2 of 3 quantiles (*p* < 0.05). No regional effects were observed.

### Heart failure 30-day readmission rates

Higher rates of hospice utilization were associated with lower 30-day readmission rates for heart failure in the 50th percentile (median) only (*p* = 0.01), as seen in Additional file 1: Table S5.

A greater number of acute care beds available per 1000 residents was associated with higher readmission rates in all 3 quantiles (*p* < 0.05), as were greater median income and higher population density (*p* < 0.05). Higher quality of care scores were not associated with heart failure readmission rates. The Northeast and Southern regions had higher rates of readmission in comparison to the Midwestern region.

More detail regarding each of the above quantile regressions is available in the Additional file 1: Table S6-S9.

### Effect size

For both pneumonia mortality and readmission, a change in hospice utilization sufficient to move from the 25th to the 75th percentile would be associated with a change in pneumonia mortality or readmission rates equivalent to at most 5% relative to other reporting hospitals.

### Sensitivity analysis

**Fig. 2:**
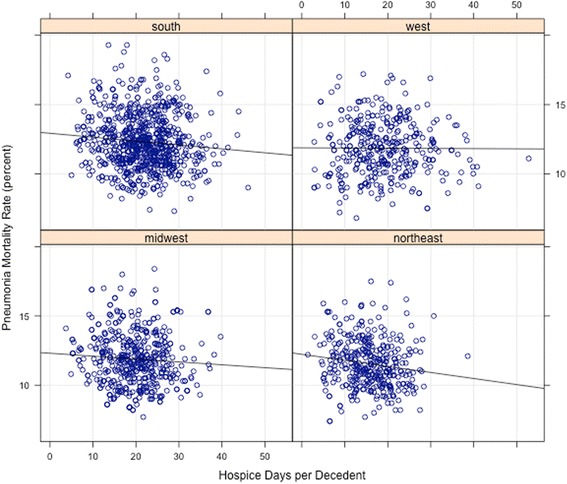
Hospice utilization and pneumonia mortality rates for the four regions of the United States

Higher rates of hospice utilization were associated with lower 30-day mortality and readmission rates for pneumonia as well as lower 30-day readmission rates for heart failure (*p* < 0.05 for all) in a multilevel model (random intercept, grouped by region) performed as a sensitivity analysis. Lattice plots of regional hospice utilization and publicly reported pneumonia mortality rates are available in Fig. 2, with further detail regarding each of the multilevel models in the Additional file 1: Table S10-S13.

## Discussion

Among hospitals reporting both hospice utilization and CMS outcomes during 2012, those hospitals with higher rates of hospice utilization reported lower 30-day mortality and readmission rates for pneumonia. These associations persisted after controlling for quality of care. These results raise questions about whether and to what extent patients’ choices to undertake less aggressive medical therapy might influence the hospital-level outcome rates reported by CMS.

Importantly, the two major outcome measures (30-day mortality and 30-day hospital readmission) handle hospice status differently. Hospital 30-day mortality rates exclude patients enrolled in hospice before the second hospital day, while 30-day readmission rates include hospice patients.

Our finding that hospitals with a greater proportion of patients enrolled in hospice have lower CMS-reported 30-day mortality rates for pneumonia suggests that the exclusion of these patients may have introduced an artifact in hospital-level reported 30-day mortality rates. This is likely caused by an interaction between the severity of illness risk-adjustment performed by CMS and higher mortality seen in hospice patients that is not captured by that CMS risk-adjustment algorithm (based as it is on data abstracted from billing codes) [6]. If preferentially high-risk patients are systematically excluded from certain hospitals at a higher rate, it then follows that those same hospitals might similarly report an artifactually lower 30-day mortality rate than comparable hospitals with a lower proportion of excluded high-risk (i.e., hospice-enrolled) patients. Our findings are compatible with, although not proof of, such a mechanism, among patients with pneumonia.

Readmission rates are calculated differently; hospice patients are included in these measures. If patients enrolled in hospice have a lower risk of readmission (based on a likely desire to die at home or another non-hospital setting) [20], then hospitals with higher rates of hospice utilization should have lower readmission rates, which is, again, what we observed for pneumonia.

Such associations could also represent a measure of quality that is not captured in the standard quality metrics (i.e., hospitals with higher hospice utilization also provide better care not measured by current metrics or communities that support hospice measures have distinct demographics, as may be the case in La Crosse, Wisconsin) or it could represent the above methods of accounting for patients on hospice. No matter the mechanism, this could create a moral hazard, an incentive for healthcare facilities to encourage hospice use independent of individual patients’ desires for it.

In other words, CMS-reported outcomes may inadvertently incentivize certain institutional or community initiatives [21], that lead to higher rates of hospice utilization independent of patient characteristics and/or preferences. That payments to hospitals by CMS are also partly linked to performance on these CMS-reported outcome rates (via the Value Based Purchasing program) adds a complex financial component to this discussion. Whether this specific incentive shapes hospice strategy at the community or hospital level is not known: we do not believe such a moral hazard is responsible for the main associations found in our study.

Pneumonia is an important disease state relevant to questions about end-of-life decision making, as it is frequently the cause of death in vulnerable patients with multiple comorbidities towards the end of life [22, 23]. No consistent effect of hospice utilization was observed for heart failure mortality and readmission in our primary analysis. Whether this reflects a difference in the mechanism of death, the underlying CMS risk-adjustment algorithms (the heart failure readmission model has very limited predictive accuracy) [24], the availability of “rescue” therapies for advanced heart failure [25], the longitudinal relationship with a heart failure team, or perhaps masking of an effect by collinearity with other covariates is unknown.

The effect size in our study was modest, but we believe that it likely underestimates the true effect size. Our analysis used matched hospital data, but the CMS-reported outcomes were condition-specific, whereas hospice utilization was reported by the Dartmouth Atlas using patients with any of a panel of chronic illnesses [12]. The populations are not strictly overlapping, even as they come from the same hospitals. We suspect that condition-specific hospice utilization would confirm our findings, with a more robust effect size, by decreasing experimental noise from unrelated patient populations. A sensitivity analysis, using a multilevel model confirmed the main findings of this study.

Our findings also add to the study of resource utilization and healthcare preferences, which have reported mixed results in terms of reducing readmission rates and other markers of healthcare utilization following interventions designed to increase the use of palliative care services (including hospice) [26–29]. The amount of hospice utilization observed in our study is consistent with previously published results and trends [30]. We also observed that hospitals with higher quality of care scores had lower 30-day mortality rates for both pneumonia and heart failure, consistent with a 2006 study of CMS measures [2], and a multicenter study of Medicare patients in 1997 [31].

Our analysis also found an association between acute care bed availability and readmission rates, echoing a 1994 study of hospitals in Boston and New Haven that found readmission rates after inpatient treatment for one of five conditions was 60% higher in Boston, a finding not explained by severity of illness [32]. Say’s Law of classical economics [33], ‘supply creates its own demand’, may apply to this association between bed availability and readmission rates, independent of patient outcomes or preferences [34, 35].

Other researchers have also identified unexpected interactions between publicly reported outcomes and patient-level factors associated with end-of-life decision making. In a study of California inpatients with pneumonia, higher DNR (Do Not Resuscitate, an advance directive that precludes resuscitation in the event of cardiac arrest) rates were associated with higher mortality rates. However, after adjusting for patient DNR status and inter-hospital variability in DNR rates, those hospitals with higher DNR rates were found to have lower pneumonia mortality rates, suggesting that taking this information into account could lead to more accurate reporting [36].

Our findings have limitations. Unmeasured confounding could introduce bias, although we controlled for quality of care, geographical region, and acute care bed availability as well as more local effects such as median income and population density. The exact metric of hospice utilization, mean number of days in hospice per decedent for a given hospital, was chosen as it reflected both duration and proportion of hospice utilization. As the unit of study was the hospital, our results cannot necessarily be generalized to other levels of measurement, such as individual patients or larger geographic associations. No adjustment was made for the multiple comparisons made in this study.

## Conclusion

In conclusion, we present evidence that higher rates of hospice utilization are associated with lower 30-day pneumonia mortality and readmission rates as reported by CMS. The extent to which this association represents or fails to represent the values and priorities of individual patients and their families deserves further study to be sure that regulatory incentives appropriately align with patient-centered medical care.

## Additional file

Additional file 1: Online Data Supplement. **Table S1.** Chronic conditions in the dartmouth atlas chronic illness cohort. **Table S2.** Inpatient quality indicators collected by the center for medicare and medicaid services, 2012. **Table S3.** Hospital characteristics by quantile of hospice utilization. **Table S4.** Quantile regression of heart failure 30-day mortality rates. **Table S5.** Quantile regression of heart failure 30-day readmission rates. **Table S6.** Pneumonia mortality quantile regression detailed results. **Table S7.** Pneumonia readmission quantile regression detailed results. **Table S8.** Heart failure mortality quantile regression detailed results. **Table S9.** Heart failure readmission quantile regression detailed results. **Table S10.** Pneumonia mortality multi-level model. **Table S11.** Pneumonia readmission multi-level model. **Table S12.** Heart failure mortality multi-level model. **Table S13.** Heart failure readmission multi-level model. (DOCX 42 kb)

## Abbreviations

CMS: Center for medicare and medicaid services
DNR: Do not resuscitate
HQA: Hospital quality alliance
US: United States
