# Supplementary material for: Association of hospice utilization and publicly reported outcomes following hospitalization for pneumonia or heart failure: a retrospective cohort study
Source: BMC Health Serv Res. 2018 Jan 9;18:12. doi: 10.1186/s12913-017-2801-3 (PMC5761109; doi:10.1186/s12913-017-2801-3)
Supplement: Additional file 1: — Online Data Supplement. Table S1. Chronic conditions in the dartmouth atlas chronic illness cohort. Table S2. Inpatient quality indicators collected by the center for medicare and medicaid services, 2012. Table S3. Hospital characteristics by quantile of hospice utilization. Table S4. Quantile regression of heart failure 30-day mortality rates. Table S5. Quantile regression of heart failure 30-day readmission rates. Table S6. Pneumonia mortality quantile regression detailed results. Table S7. Pneumonia readmission quantile regression detailed results. Table S8. Heart failure mortality quantile regression detailed results. Table S9. Heart failure readmission quantile regression detailed results. Table S10. Pneumonia mortality multi-level model. Table S11. Pneumonia readmission multi-level model. Table S12. Heart failure mortality multi-level model. Table S13. Heart failure readmission multi-level model. (DOCX 42 kb) [file 12913_2017_2801_MOESM1_ESM.docx]

Association of hospice utilization and publicly reported outcomes following hospitalization for pneumonia or heart failure

Soowhan Lah MD^1,2,3^, Emily L. Wilson MS^1,2^, Sarah Beesley MD^1,2,3^, Iftach Sagy MD^4^, James Orme MD^1,2,3^,Victor Novack MD PhD^4,5^, Samuel M. Brown MD MS^1,2,3^

^1^Center for Humanizing Critical Care, Intermountain Healthcare, Murray, UT

^2^Pulmonary and Critical Care Medicine, Intermountain Medical Center, Murray, UT

^3^Pulmonary and Critical Care Medicine, University of Utah School of Medicine, SLC, UT

^4^Clinical Research Center, Soroka University Medical Center, Israel

^5^Beth Israel Deaconess Medical Center and Harvard Medical School, Boston, MA

**Corresponding author**: Samuel M. Brown, Shock Trauma ICU, Intermountain Medical Center, 5121 S. Cottonwood Street, Murray, UT 84107, O: (801) 507-6529, F: (801) 507-5578

Contributions to manuscript: Conception (SMB, SL), data acquisition (SL), data analysis (SL, EW, IS, VN, SMB), writing manuscript (SL, SMB), revising manuscript for important intellectual content (all authors), approval of final copy (all authors)

Online Data Supplement

Appendix 1. Additional methods

Detailed statistical Methods

Linear regression is a method by which a line of best fit is described for response variable *y* as a condition of predictor variable X, *y* = f(Χ). Linear regression uses this function to estimate rates of change to the mean of the response variable.

We chose quantile regression for its robustness in the analysis of non-normally distributed data with varying degrees of heterogeneity.^2^ Quantile regression provides the advantage of several regression equations being fit to specific components of the distribution of the response variable, allowing for a more complete portrayal of the predictor-response relationship, which may include non-linearities. In this paper the term ‘percentiles’ was used for ease of understanding, though ‘quantile’ is the technical term. Quantile regression allows the various percentiles (or quantiles) of a response distribution to be modeled.^1^ In other words, for example, a regression equation may be fitted to the 25^th^ percentile of the response variable (rather than the mean of the response variable).

References

1. Cade BS. Estimating equivalence with quantile regression. *Ecol. Appl.* Jan 2011;21(1):281-289.

2. Koenker R. Quantile Regression in R: A Vignette. 2015; https://cran.r-project.org/web/packages/quantreg/vignettes/rq.pdf. Accessed May 23, 2016.

Appendix 2. Additional results

Table S1. Chronic Conditions in the Dartmouth Atlas Chronic Illness Cohort

| Primary Chronic Condition | 2003–07 Hospital-Specific Chronic Illness Cohort |
| --- | --- |
|  | Number of Decedents |
| Malignant Cancer/Leukemia | 809,926 |
| Congestive heart failure | 1,497,465 |
| Chronic Pulmonary disease | 903,696 |
| Dementia | 570,479 |
| Diabetes with end organ damage | 48,342 |
| Peripheral vascular disease | 108,696 |
| Chronic renal failure | 349,611 |
| Severe Chronic Liver disease | 52,841 |
| Coronary artery disease | 325,152 |
| Total Decedents | 4,666,208 |

Table S2. Inpatient Quality Indicators collected by the Center for Medicare and Medicaid Services, 2012

[1] "Average number of minutes before outpatients with chest pain or possible heart attack got an ECG A lower number of minutes is better"

[2] "Average number of minutes before outpatients with chest pain or possible heart attack who needed specialized care were transferred to another hospital A lower number of minutes is better"

[3] "Children and their caregivers who received a home management plan of care document while hospitalized for asthma Higher percentages are better"

[4] "Children who received reliever medication while hospitalized for asthma Higher percentages are better"

[5] "Children who received systemic corticosteroid medication (oral and IV medication that reduces inflammation and controls symptoms) while hospitalized for asthma Higher percentages are better"

[6] "Heart attack patients given a prescription for a statin at discharge Higher percentages are better"

[7] "Heart attack patients given aspirin at discharge Higher percentages are better"

[8] "Heart attack patients given fibrinolytic medication within 30 minutes of arrival Higher percentages are better"

[9] "Heart attack patients given PCI within 90 minutes of arrival Higher percentages are better"

[10] "Heart failure patients given ACE inhibitor or ARB for Left Ventricular Systolic Dysfunction (LVSD) Higher percentages are better"

[11] "Heart failure patients given an evaluation of Left Ventricular Systolic (LVS) function Higher percentages are better"

[12] "Heart failure patients given discharge instructions Higher percentages are better"

[13] "Heart surgery patients whose blood sugar (blood glucose) is kept under good control in the days right after surgery Higher percentages are better"

[14] "Median Time to Fibrinolysis"

[15] "Outpatients having surgery who got an antibiotic at the right time (within one hour before surgery) Higher percentages are better"

[16] "Outpatients having surgery who got the right kind of antibiotic Higher percentages are better"

[17] "Outpatients with chest pain or possible heart attack who got aspirin within 24 hours of arrival Higher percentages are better"

[18] "Outpatients with chest pain or possible heart attack who got drugs to break up blood clots within 30 minutes of arrival Higher percentages are better"

[19] "Patients having surgery who were actively warmed in the operating room or whose body temperature was near normal by the end of surgery Higher percentages are better"

[20] "Patients who got treatment at the right time (within 24 hours before or after their surgery) to help prevent blood clots after certain types of surgery Higher percentages are better"

[21] "Pneumonia patients given the most appropriate initial antibiotic(s) Higher percentages are better"

[22] "Pneumonia patients whose initial emergency room blood culture was performed prior to the administration of the first hospital dose of antibiotics Higher percentages are better"

[23] "Surgery patients who were given an antibiotic at the right time (within one hour before surgery) to help prevent infection Higher percentages are better"

[24] "Surgery patients who were given the right kind of antibiotic to help prevent infection Higher percentages are better"

[25] "Surgery patients who were taking heart drugs called beta blockers before coming to the hospital, who were kept on the beta blockers during the period just before and after their surgery Higher percentages are better"

[26] "Surgery patients whose doctors ordered treatments to prevent blood clots after certain types of surgeries Higher percentages are better"

[27] "Surgery patients whose preventive antibiotics were stopped at the right time (within 24 hours after surgery) Higher percentages are better"

[28] "Surgery patients whose urinary catheters were removed on the first or second day after surgery Higher percentages are better"

Table S3. Hospital Characteristics by Quantile of Hospice Utilization

| Quantile | Days on Hospice per Decedent | Quality Score (out of 100) | Median Income (in thousands, USD) | Acute care beds per 1000 residents |
| --- | --- | --- | --- | --- |
| 1 | 10.9 | 94.2 | 50.2 | 2.26 |
| 2 | 17.3 | 95.6 | 50.2 | 2.26 |
| 3 | 21.8 | 95.9 | 53.0 | 2.12 |
| 4 | 28.6 | 95.6 | 48.8 | 2.19 |

Table S4. Quantile Regression of Heart Failure 30-day Mortality Rates

|  | 25^th^ percentile | 50^th^ percentile | 75^th^ percentile |
| --- | --- | --- | --- |
| Hospice days per decedent | **0.014** | -0.0016 | -0.012 |
| Quality of care | **-0.039** | **-0.034** | **-0.037** |
| Acute care hospital beds per 1,000 residents | -0.11 | **-0.18** | -0.078 |
| Median income (in thousands, USD) | -0.0038 | **--0.0087** | **-0.010** |
| Population density (in thousands per square mile) | **-.062** | **-0.065** | **-0.036** |
| Northeast region | 0.098 | -0.18 | -0.22 |
| Southern region | 0.12 | 0.016 | -0.071 |
| Western region | 0.16 | 0.097 | 0.20 |

*Values for which p < 0.05 are **bolded**

**Reference region: Midwest

Table S5. Quantile Regression of Heart Failure 30-day Readmission Rates

|  | 25^th^ percentile | 50^th^ percentile | 75^th^ percentile |
| --- | --- | --- | --- |
| Hospice days per decedent | -0.013 | **-0.018** | -0.015 |
| Quality of care | -0.016 | -0.0049 | -0.0020 |
| Acute care hospital beds per 1,000 residents | **0.53** | **0.68** | **0.63** |
| Median income (in thousands, USD) | **0.011** | **0.010** | **0.063** |
| Population density (in thousands, per square mile) | **0.049** | **0.062** | **0.065** |
| Northeast region | **0.72** | **0.91** | **0.93** |
| Southern region | **0.34** | **0.45** | **0.56** |
| Western region | **-0.32** | -0.12 | 0.0064 |

*Values for which p < 0.05 are **bolded**

**Reference region: Midwest

Table S6 Pneumonia Mortality Quantile Regression Detailed Results

Overall Model

Coefficients:

25th 50th 75th

percentile percentile percentile

(Intercept) 15.9236504503 16.423584574 20.347848559

Hospice days per decedent -0.0160345082 -0.027953461 -0.022934889

Quality -0.0471774411 -0.036603683 -0.064335871

Acute Care Hospital Beds per 1,000 Residents -0.0098666469 -0.001039979 -0.049566531

Median income -0.0000086718 -0.000010371 -0.000012000

Population density -0.0000405353 -0.000031464 -0.000032868

Northeast region -0.0842324945 -0.212787278 -0.137496403

Southern region 0.3973665217 0.280608116 0.377935825

Western region 0.1488394850 0.104541863 0.286245558

Degrees of freedom: 2061 total; 2052 residual

25th percentile

Coefficients:

Value Std. Error t value Pr(>|t|)

(Intercept) 15.92365 1.07369 14.83074 0.00000

Hospice days per decedent -0.01603 0.00674 -2.37858 0.01747

Quality -0.04718 0.01063 -4.43635 0.00001

Acute Care Hospital Beds per 1,000 Residents -0.00987 0.08107 -0.12170 0.90315

Median income -0.00001 0.00000 -3.62308 0.00030

Population density -0.00004 0.00002 -2.39350 0.01678

Northeast region -0.08423 0.15058 -0.55939 0.57596

Southern region 0.39737 0.11119 3.57379 0.00036

Western region 0.14884 0.18172 0.81906 0.41285

tau: [1] 0.5

Coefficients:

Value Std. Error t value Pr(>|t|)

(Intercept) 16.42358 1.25442 13.09253 0.00000

Hospice days per decedent -0.02795 0.00697 -4.01199 0.00006

Quality -0.03660 0.01255 -2.91563 0.00359

Acute Care Hospital Beds per 1,000 Residents -0.00104 0.08130 -0.01279 0.98980

Median income -0.00001 0.00000 -4.40058 0.00001

Population density -0.00003 0.00001 -6.26500 0.00000

Northeast region -0.21279 0.14896 -1.42844 0.15332

Southern region 0.28061 0.10981 2.55535 0.01068

Western region 0.10454 0.15008 0.69658 0.48615

75th percentile

Coefficients:

Value Std. Error t value Pr(>|t|)

(Intercept) 20.34785 1.98922 10.22905 0.00000

Hospice days per decedent -0.02293 0.01115 -2.05659 0.03985

Quality -0.06434 0.02019 -3.18726 0.00146

Acute Care Hospital Beds per 1,000 Residents -0.04957 0.13361 -0.37097 0.71070

Median income -0.00001 0.00000 -3.40328 0.00068

Population density -0.00003 0.00001 -2.61370 0.00902

Northeast region -0.13750 0.19164 -0.71748 0.47316

Southern region 0.37794 0.18079 2.09046 0.03670

Western region 0.28625 0.20696 1.38308 0.16679

Table S7. Pneumonia Readmission Quantile Regression Detailed Results

Overall Model

Coefficients:

25th 50th 75th

percentile percentile percentile

(Intercept) 17.1263102843 16.6674171096 15.2250578232

Hospice days per decedent -0.0146844535 -0.0208261379 -0.0173862091

Quality -0.0048753215 0.0137685480 0.0350080639

Acute Care Hospital Beds per 1,000 Residents 0.2465719993 0.2920263373 0.4963529878

Median income 0.0000072965 0.0000033634 0.0000036623

Population density 0.0000364295 0.0000403132 0.0000481457

Northeast region 0.2586606986 0.1414771833 0.2000395550

Southern region 0.2523287283 0.1303327815 0.2576647053

Western region -0.3915347415 -0.5357852982 -0.4149994924

Degrees of freedom: 2062 total; 2053 residual

25th percentile

Coefficients:

Value Std. Error t value Pr(>|t|)

(Intercept) 17.12631 0.89269 19.18507 0.00000

Hospice days per decedent -0.01468 0.00621 -2.36440 0.01815

Quality -0.00488 0.00841 -0.57995 0.56201

Acute Care Hospital Beds per 1,000 Residents 0.24657 0.08278 2.97874 0.00293

Median income 0.00001 0.00000 2.64571 0.00821

Population density 0.00004 0.00000 8.45981 0.00000

Northeast region 0.25866 0.14628 1.76831 0.07716

Southern region 0.25233 0.12990 1.94242 0.05222

Western region -0.39153 0.15817 -2.47547 0.01339

50th percentile

Coefficients:

Value Std. Error t value Pr(>|t|)

(Intercept) 16.66742 0.72800 22.89481 0.00000

Hospice days per decedent -0.02083 0.00687 -3.03097 0.00247

Quality 0.01377 0.00686 2.00767 0.04481

Acute Care Hospital Beds per 1,000 Residents 0.29203 0.07529 3.87856 0.00011

Median income 0.00000 0.00000 1.36332 0.17293

Population Population density 0.00004 0.00001 4.65531 0.00000

Northeast region 0.14148 0.15725 0.89970 0.36839

Southern region 0.13033 0.12141 1.07348 0.28318

Western region -0.53579 0.14834 -3.61177 0.00031

75th percentile

Coefficients:

Value Std. Error t value Pr(>|t|)

(Intercept) 15.22506 1.16397 13.08033 0.00000

Hospice days per decedent -0.01739 0.00825 -2.10698 0.03524

Quality 0.03501 0.01189 2.94361 0.00328

Acute Care Hospital Beds per 1,000 Residents 0.49635 0.08120 6.11272 0.00000

Median income 0.00000 0.00000 1.20163 0.22965

Population density 0.00005 0.00000 11.94000 0.00000

Northeast region 0.20004 0.17088 1.17065 0.24187

Southern region 0.25766 0.15651 1.64630 0.09985

Western region -0.41500 0.18744 -2.21405 0.02693

Table S8. Heart Failure Mortality Quantile Regression Detailed Results

Overall Model

Coefficients:

25th 50th 75th

percentile percentile percentile

(Intercept) 14.4312258361 15.8012265731 17.2117569767

Hospice days per decedent 0.0135331636 -0.0016034778 -0.0114891398

Quality -0.0386141951 -0.0343739890 -0.0373852340

Acute Care Hospital Beds per 1,000 Residents -0.1070363679 -0.1747310761 -0.0779920275

Median income -0.0000037891 -0.0000087224 -0.0000099593

Population density -0.0000624384 -0.0000648481 -0.0000358825

Northeast region 0.0983566238 -0.1788987522 -0.2224447712

Southern region 0.1226674987 0.0160098304 -0.0710835217

Western region 0.1621683032 0.0974434203 0.2008973393

Degrees of freedom: 2060 total; 2051 residual

25th percentile

Coefficients:

Value Std. Error t value Pr(>|t|)

(Intercept) 14.43123 1.02445 14.08678 0.00000

Hospice days per decedent 0.01353 0.00544 2.48752 0.01294

Quality -0.03861 0.01003 -3.85160 0.00012

Acute Care Hospital Beds per 1,000 Residents -0.10704 0.06976 -1.53433 0.12510

Median income 0.00000 0.00000 -1.63163 0.10291

Population density -0.00006 0.00001 -6.25934 0.00000

Northeast region 0.09836 0.12012 0.81879 0.41300

Southern region 0.12267 0.10113 1.21300 0.22527

Western region 0.16217 0.12068 1.34384 0.17915

50th percentile

Coefficients:

Value Std. Error t value Pr(>|t|)

(Intercept) 15.80123 1.15361 13.69714 0.00000

Hospice days per decedent -0.00160 0.00644 -0.24912 0.80329

Quality -0.03437 0.01188 -2.89228 0.00386

Acute Care Hospital Beds per 1,000 Residents -0.17473 0.06242 -2.79933 0.00517

Median income -0.00001 0.00000 -5.35008 0.00000

Population density -0.00006 0.00001 -5.70336 0.00000

Northeast region -0.17890 0.11752 -1.52230 0.12809

Southern region 0.01601 0.10100 0.15851 0.87407

Western region 0.09744 0.14002 0.69594 0.48654

75th percentile

Coefficients:

Value Std. Error t value Pr(>|t|)

(Intercept) 17.21176 1.56072 11.02808 0.00000

Hospice days per decedent -0.01149 0.00853 -1.34684 0.17818

Quality -0.03739 0.01545 -2.41904 0.01565

Acute Care Hospital Beds per 1,000 Residents -0.07799 0.12177 -0.64046 0.52194

Median income -0.00001 0.00000 -3.92394 0.00009

Population density -0.00004 0.00001 -5.24004 0.00000

Northeast region -0.22244 0.17652 -1.26015 0.20776

Southern region -0.07108 0.13867 -0.51261 0.60828

Western region 0.20090 0.18519 1.08481 0.27813

Table S9. Heart Failure Readmission Quantile Regression Detailed Results

Overall Model

Coefficients:

25th 50th 75th

percentile percentile percentile

(Intercept) 23.161186610 23.018691074 24.1812632943

Hospice days per decedent -0.013174241 -0.017559434 -0.0149170129

Quality -0.015912327 -0.004849804 -0.0020013971

Acute Care Hospital Beds per 1,000 Residents 0.527126502 0.674814968 0.6332834992

Median income 0.000011374 0.000010348 0.0000062692

Population density 0.000048500 0.000062346 0.0000650667

Northeast region 0.721661379 0.913554508 0.9268515212

Southern region 0.334831941 0.452706761 0.5574284667

Western region -0.320521264 -0.114552511 0.0063856843

Degrees of freedom: 2060 total; 2051 residual

25th percentile

Coefficients:

Value Std. Error t value Pr(>|t|)

(Intercept) 23.16119 0.87968 26.32897 0.00000

Hospice days per decedent -0.01317 0.00833 -1.58197 0.11381

Quality -0.01591 0.00952 -1.67065 0.09494

Acute Care Hospital Beds per 1,000 Residents 0.52713 0.06755 7.80352 0.00000

Median income 0.00001 0.00000 3.82982 0.00013

Population density 0.00005 0.00001 5.65379 0.00000

Northeast region 0.72166 0.20053 3.59877 0.00033

Southern region 0.33483 0.14489 2.31089 0.02094

Western region -0.32052 0.15846 -2.02273 0.04323

50th percentile

Coefficients:

Value Std. Error t value Pr(>|t|)

(Intercept) 23.01869 0.93481 24.62395 0.00000

Hospice days per decedent -0.01756 0.00714 -2.45835 0.01404

Quality -0.00485 0.00907 -0.53489 0.59278

Acute Care Hospital Beds per 1,000 Residents 0.67481 0.08473 7.96385 0.00000

Median income 0.00001 0.00000 3.41188 0.00066

Population density 0.00006 0.00001 5.24893 0.00000

Northeast region 0.91355 0.16212 5.63521 0.00000

Southern region 0.45271 0.12370 3.65986 0.00026

Western region -0.11455 0.16299 -0.70281 0.48225

75th percentile

Coefficients:

Value Std. Error t value Pr(>|t|)

(Intercept) 24.18126 0.87665 27.58381 0.00000

Hospice days per decedent -0.01492 0.00853 -1.74844 0.08054

Quality -0.00200 0.00954 -0.20985 0.83380

Acute Care Hospital Beds per 1,000 Residents 0.63328 0.05299 11.95126 0.00000

Median income 0.00001 0.00000 2.72392 0.00651

Population density 0.00007 0.00001 12.29566 0.00000

Northeast region 0.92685 0.17603 5.26545 0.00000

Southern region 0.55743 0.15171 3.67440 0.00024

Western region 0.00639 0.17960 0.03556 0.97164

Table S10. Pneumonia Mortality Multi-Level Model

Random effects:

Formula: ~1 | region

(Intercept) Residual

StdDev: 0.21426 1.8409

Fixed effects: rate ~ Hospice.days.per.decedent + Quality +

Acute Care Hospital Beds per 1,000 Residents + Median Income + Population Density

Value Std.Error DF t-value p-value

(Intercept) 17.2680 0.94554 2047 18.2627 0.0000

Hospice.days.per.decedent -0.0199 0.00618 2047 -3.2161 0.0013

Quality -0.0447 0.00972 2047 -4.6014 0.0000

Acute Care Hospital Beds per 1,000 Residents -0.0102 0.05110 2047 -0.2005 0.8411

Median income 0.0000 0.00000 2047 -4.9854 0.0000

Population Density 0.0000 0.00000 2047 -6.3879 0.0000

Table S11. Pneumonia Readmission Multi-Level Model

Random effects:

Formula: ~1 | region

(Intercept) Residual

StdDev: 0.3307 1.5973

Fixed effects: rate ~ Hospice.days.per.decedent + Quality +

Acute Care Hospital Beds per 1,000 Residents + Median Income + Population Density

Value Std.Error DF t-value p-value

(Intercept) 17.6158 0.83183 2048 21.1771 0.0000

Hospice.days.per.decedent -0.0199 0.00538 2048 -3.7002 0.0002

Quality 0.0065 0.00844 2048 0.7686 0.4422

Acute Care Hospital Beds per 1,000 Residents 0.2166 0.04456 2048 4.8618 0.0000

Median income 0.0024 0.00191 2048 1.2588 0.2082

Population Density 0.0369 0.00431 2048 8.5730 0.0000

Table S12. Heart Failure Mortality Multi-Level Model

Random effects:

Formula: ~1 | region

(Intercept) Residual

StdDev: 0.021729 1.5896

Fixed effects: rate ~ Hospice.days.per.decedent + weighted + Acute.Care.Hospital.Beds.per.1.000.Residents..2012. + median + density

Value Std.Error DF t-value p-value

(Intercept) 15.878 0.8102 2046 19.5982 0.0000

Hospice.days.per.decedent -0.002 0.0051 2046 -0.3656 0.7147

weighted -0.037 0.0084 2046 -4.4195 0.0000

Acute.Care.Hospital.Beds.per.1.000.Residents..2012. -0.106 0.0424 2046 -2.4921 0.0128

median -6.677 1.8719 2046 -3.5673 0.0004

density -47.466 4.1888 2046 -11.3318 0.0000

Table S13. Heart Failure Readmission Multi-Level Model

Random effects:

Formula: ~1 | region

(Intercept) Residual

StdDev: 0.43221 1.8506

Fixed effects: rate ~ Hospice.days.per.decedent + weighted + Acute.Care.Hospital.Beds.per.1.000.Residents..2012. + median + density

Value Std.Error DF t-value p-value

(Intercept) 24.6828 0.96897 2046 25.4732 0.0000

Hospice.days.per.decedent -0.0200 0.00624 2046 -3.2010 0.0014

weighted -0.0090 0.00977 2046 -0.9232 0.3560

Acute.Care.Hospital.Beds.per.1.000.Residents..2012. 0.4044 0.05167 2046 7.8270 0.0000

median 0.0052 0.00221 2046 2.3317 0.0198

density 0.0485 0.00499 2046 9.7177 0.0000
